# Diverse *Kinetoplastida* detected in fecal samples from wild non-human primates across three African regions

**DOI:** 10.1016/j.ijppaw.2026.101277

**Published:** 2026-08-17

**Authors:** Charles Kumakamba, Inestin Amona, Clément Labarrere, Aku Elomou Essenou, Georges Diatta, Cheikh Sokhna, Jean Akiana, Jean-Jacques Muyembe-Tamfum, Florence Fenollar, Oleg Mediannikov

**Affiliations:** a Microbes Evolution Phylogénie et Infection (MEPHI), Aix Marseille Univ, AP-HM, Marseille, France; b Institut Hospitalo-Universitaire Méditerranée Infection (IHU-MI), Marseille, France; c Institut National de Recherche Biomédicale (INRB), Democratic Republic of the Congo; d Risques Infectieux Tropicaux Microorganismes Emergents (RITMES), Aix-Marseille Univ, AP-HM, SSA, Marseille, France; e IRD, Marseille, France; f Faculté des Sciences et Techniques, Université Marien NGOUABI, Brazzaville, Republic of the Congo; g Laboratoire de Santé Publique, Brazzaville, Republic of the Congo; h RITMES, Campus International Commun UCAD-IRD of Hann, IRD, Dakar, Senegal

**Keywords:** Kinetoplastida, Non-human primates, Fecal samples, Molecular detection, Phylogenetic analysis, One health

## Abstract

**Background:**

The order *Kinetoplastida* comprises medically important parasites as well as numerous non-trypanosomatid lineages whose diversity and ecology remain poorly understood. Although studies in non-human primates (NHPs) have mainly focused on pathogenic trypanosomatids, little is known about the broader diversity of Kinetoplastida associated with wildlife. The present study aimed to characterize the diversity of Kinetoplastida detected in wild NHPs across Africa.

**Methods:**

We analyzed 884 fecal samples collected from wild NHPs in three African countries (Democratic Republic of the Congo, Republic of the Congo and Senegal). Following nucleic acid extraction, samples were screened for Kinetoplastida using a broad-range quantitative PCR targeting the 28S rRNA gene. Positive samples were subjected to conventional PCR, sequencing and phylogenetic analysis.

**Results:**

Kinetoplastida DNA was detected in 88 fecal samples from all study sites, with detection frequencies varying from 3.69% in the Democratic Republic of the Congo to 16.24% in the Republic of the Congo and 34.78% in Senegal. Phylogenetic analyses revealed a broad diversity of kinetoplastid lineages, predominantly affiliated with bodonid-related taxa, including clades closely related to *Bodo saltans*, *Parabodo nitrophilus* and *Neobodo* spp. One sequence clustered within a clade closely related to *Dimastigella trypaniformis*, whereas another group of sequences formed a clade related to *Parabodo caudatus*. A limited number of sequences also showed phylogenetic proximity to Trypanosomatidae lineages related to Trypanosoma, Leishmania and Leptomonas.

**Conclusions:**

This study expands current knowledge of the diversity of Kinetoplastida detected in fecal samples from wild NHPs and highlights the value of non-invasive molecular approaches for investigating protist diversity in wildlife. The detection of Trypanosomatidae-related lineages in fecal samples represents an uncommon finding that deserves further investigation. Further studies combining complementary sample types, strain isolation and additional molecular approaches are needed to better understand the ecological significance of the detected lineages and their relationships with vertebrate hosts.

## Highlights

• Wild African primates harbor a diverse assemblage of Kinetoplastida.
• Molecular screening of 884 fecal samples revealed multiple kinetoplastid clades.
• Bodonid-related lineages predominated across study sites.
• Dimastigella- and Parabodo-related lineages were detected in several NHP species.
• Trypanosomatidae-related sequences were identified in fecal samples from NHPs.

## Graphical abstract

**Figure:**
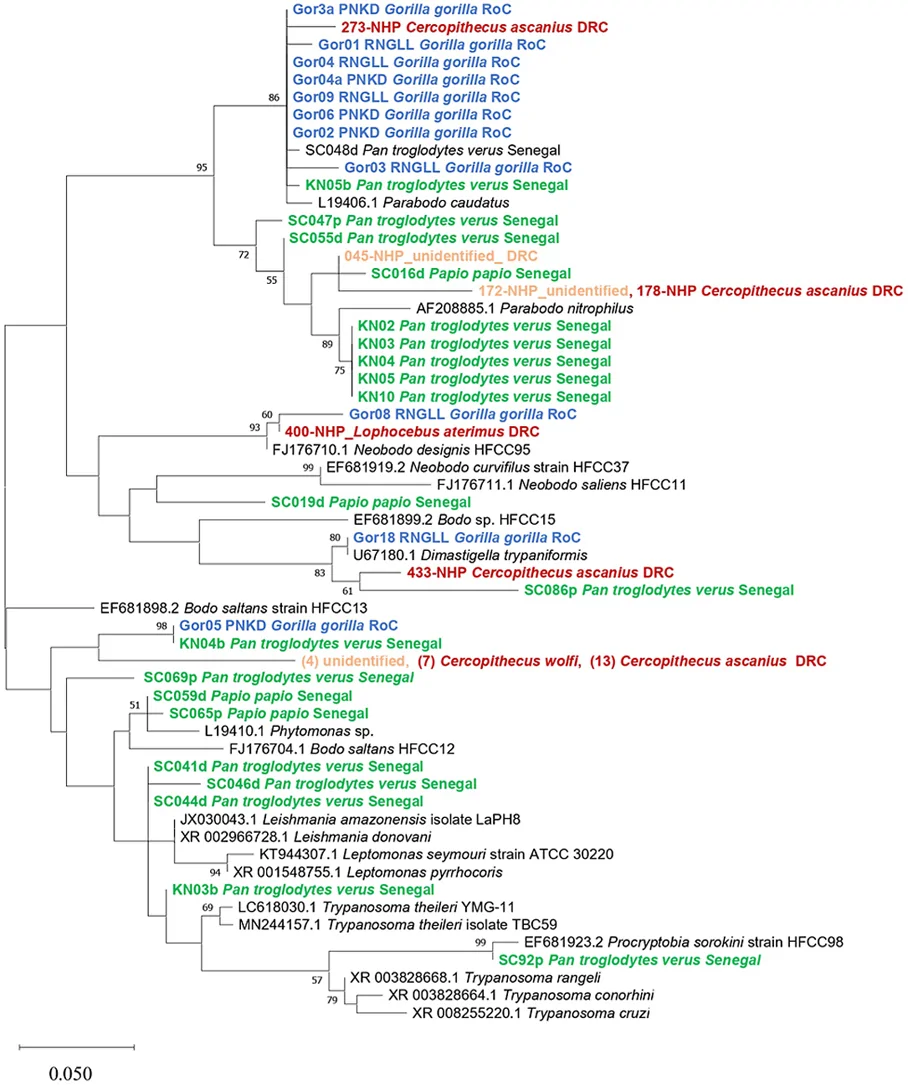

## 1 Introduction

The order *Kinetoplastida* comprises a highly diverse group of flagellated protists inhabiting aquatic, terrestrial and host-associated environments (D'avila-Levy et al., 2015; Simpson et al., 2006). Among them, the family *Trypanosomatidae* includes medically and veterinary important parasites such as Trypanosoma and Leishmania, which are responsible for major diseases affecting humans and animals worldwide (Burza et al., 2018; Franco et al., 2014; Stuart et al., 2008). In contrast, numerous non-trypanosomatid kinetoplastids, including members of the Bodonida, are generally regarded as free-living organisms, although their diversity, ecology and interactions with vertebrate hosts remain poorly understood (D'avila-Levy et al., 2015; Simpson et al., 2006). Recent environmental molecular surveys have considerably expanded our understanding of kinetoplastid diversity, revealing that many lineages remain insufficiently characterized and that their ecological roles are still largely unknown (D'avila-Levy et al., 2015; Simpson et al., 2006).

In sub-Saharan Africa, kinetoplastids of the family Trypanosomatidae are responsible for important human and animal diseases. *Trypanosoma brucei gambiense*, the causative agent of human African trypanosomiasis (HAT), remains a public health concern in several foci despite substantial progress towards disease elimination (Franco et al., 2014; Lumbala et al., 2015). Likewise, animal African trypanosomiasis caused principally by *T. congolense* and *T. vivax* continues to have a major impact on livestock production across endemic regions (Auty et al., 2015; Giordani et al., 2016). Although pathogenic trypanosomatids have been extensively investigated, considerably less attention has been devoted to the diversity of other kinetoplastid lineages circulating within African ecosystems.

Non-human primates (NHPs) are of particular interest for investigating microorganisms circulating at the wildlife-environment interface because of their close phylogenetic relationship with humans, their ecological diversity and their frequent overlap with human activities (Chapman et al., 2013; Medkour et al., 2021a; Perelman et al., 2011; Pozzi et al., 2014). Natural infections with several Trypanosoma species, including *T. brucei*, *T. congolense*, *T. vivax* and *T. theileri*, have been reported in African NHPs (Cordon-Obras et al., 2015; Herder et al., 2002; Medkour et al., 2021b). Similarly, exposure to *Leishmania* spp. has been documented in different primate species, suggesting that some NHPs may participate in the ecology of these parasites in endemic regions (André et al., 2020; De Oliveira et al., 2019). Nevertheless, previous investigations in NHPs have largely focused on pathogenic trypanosomatids, whereas the broader diversity of Kinetoplastida remains poorly documented.

Beyond parasitic trypanosomatids, increasing evidence indicates that environmental kinetoplastids may occasionally be detected in vertebrate clinical samples. Bodo-like flagellates have historically been reported in human urine (Hassall, 1859; Powell, A; Kohiyar, 1920; Sinton, 1912), while *Parabodo caudatus* was detected in the urine of a dog presenting with hematuria (Vandersea et al., 2015). More recently, *Dimastigella trypaniformis* was identified in the urine of a patient with urinary tract infection (Peña et al., 2025). Although these observations demonstrate that environmental kinetoplastids can be detected in vertebrate-derived samples, their biological significance, ecological relationships with vertebrate hosts and potential medical or veterinary relevance remain unknown. Detection alone does not demonstrate active infection or pathogenicity, and further investigations are required to clarify these associations.

Because fecal sampling provides a non-invasive approach for investigating microorganisms in wildlife, it represents a valuable tool for exploring the diversity of poorly characterized protists in natural ecosystems. Broad-range molecular approaches targeting conserved genetic markers further allow the detection of a wide diversity of kinetoplastid lineages without restricting analyses to known pathogenic taxa.

The present study aimed to characterize the diversity of Kinetoplastida DNA detected in fecal samples collected from wild NHPs across three African regions (the Democratic Republic of the Congo (DRC), the Republic of the Congo (RoC) and Senegal) using a broad-range molecular approach targeting the 28S rRNA gene. We further assessed the phylogenetic relationships of the detected sequences and discuss their possible ecological significance at the wildlife-environment interface, while acknowledging that DNA detection in fecal samples does not by itself demonstrate infection, host association or pathogenicity.

## 2 Materials and methods

### 2.1 Study site and sample collection

**Fig. 1:**
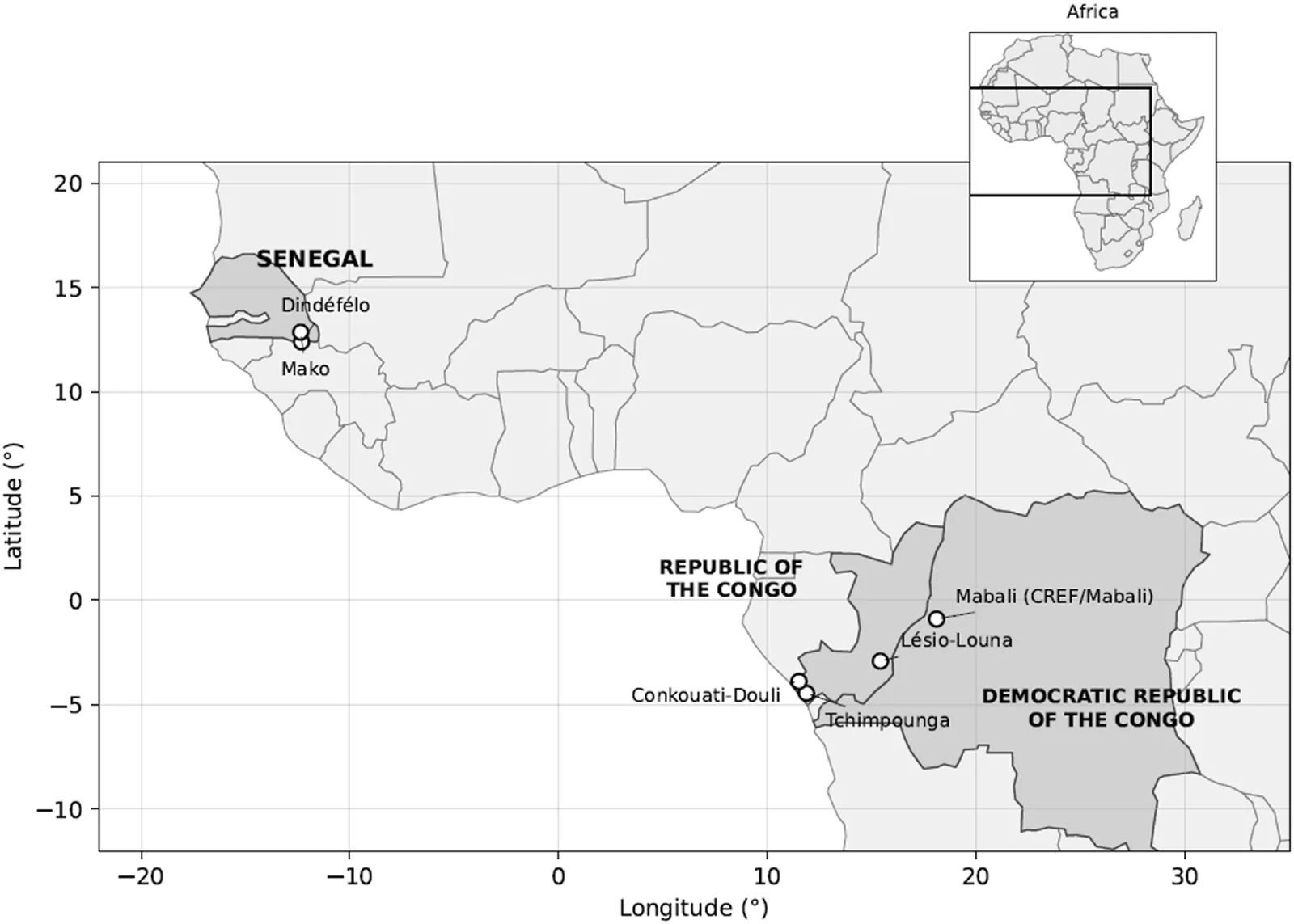
**Geographic location of the three African countries and sampling sites included in this study.***Fecal samples from wild non-human primates were collected in the Democratic Republic of the Congo (Mabali Forest Reserve), the Republic of the Congo (Tchimpounga Chimpanzee Sanctuary, Conkouati-Douli National Park, and Lésio-Louna Gorilla Natural Reserve), and Senegal (Dindéfélo and Mako). Site locations shown on the map are approximate reference locations derived from publicly available geographic information and do not represent the exact locations of individual fecal samples.****Base map: Natural Earth. Map prepared by the authors.***

A total of 884 NHPs fecal samples were collected across three countries in Central and West Africa: the DRC, the RoC, and Senegal (Fig. 1).

In the DRC, sample collection was conducted as previously described (Kumakamba et al., 2026), involving 630 fecal samples collected in December 2023 in the Mabali Forest Reserve (Équateur Province).

In Senegal, 36 samples were collected in March 2021 in the Dindéfélo region. Additional sampling was conducted in 2024, yielding 101 samples from two regions: Mako and Dindéfélo.

In the RoC, a total of 117 fecal samples were collected in 2021 and 2025. In 2021, 97 samples were collected in two protected areas located in the southern part of the country: the Tchimpounga Chimpanzee Sanctuary (n = 91) and the Conkouati-Douli National Park (PNKD) (n = 6). In 2025, an additional 20 samples were collected in the Lésio-Louna Gorilla Natural Reserve.

Fecal samples were collected from the ground in the animals’ natural environment without direct interaction, with the assistance of trackers and eco-guards. Because sampling was conducted under field conditions, the exact time elapsed between defecation and sample collection could not always be determined. Host species identification was subsequently confirmed using molecular methods.

### 2.2 Transport and storage

In the DRC, sample transport and storage were performed as previously described (Kumakamba et al., 2026), with samples maintained at −80 °C throughout the process to preserve the cold chain.

Samples collected in Senegal were transported and stored at −20 °C at the Institut de Recherche pour le Développement (IRD), Hann Maristes campus, prior to shipment to IHU-MI under appropriate cold-chain conditions.

In the RoC, fecal samples preserved either in absolute ethanol or kept fresh were initially stored at −20 °C in freezers located at the collection sites. They were subsequently transported and maintained at −20 °C at the Laboratoire National de Santé Publique in Brazzaville before being shipped to IHU-MI (Marseille, France) for molecular analyses. All required export authorizations were obtained prior to shipment.

### 2.3 Ethical authorization

The collection of NHP fecal samples was conducted in accordance with national regulations and with the appropriate research and administrative authorizations obtained in each country.

In the DRC, research authorizations have been previously described (Kumakamba et al., 2026).

In Senegal, research authorization was issued by the Ministry of Environment and Ecological Transition (No. 003924/DEFCCS/DGF, December 4, 2020).

In the RoC, research and administrative authorizations were granted by the Ministry of Scientific Research and Technological Innovation (Nos. 003/MRSIT/DGRST/DMAST and 05/MRSIT/DGRST/DMAST) and by the Congolese Agency for Wildlife and Protected Areas (ACFAP) (No. 00087/ACFAP/DG/DTS-SRM).

### 2.4 NHP sample processing, nucleic acid extraction, molecular detection of Ebola virus, verification of bacterial DNA quality, molecular identification of primate species

NHP Sample processing, nucleic acid extraction, molecular detection of Ebola virus, verification of bacterial DNA extraction quality, molecular identification of primate species were performed as previously described (Kumakamba et al., 2026).

### 2.5 Molecular detection of Kinetoplastida

Molecular screening for Kinetoplastida was performed on nucleic acids extracted from fecal samples collected across all study sites.

Detection relied on real-time quantitative PCR (qPCR) targeting the 28S gene of all Kinetoplastida. The 28S rRNA gene was selected because it enables broad-range detection of Kinetoplastida, including both trypanosomatid and non-trypanosomatid lineages, making it suitable for exploring kinetoplastid diversity in wildlife (Medkour et al., 2020). The qPCR was performed in a final volume of 20 μL containing 5 μL of template DNA, 10 μL of Roche Master Mix, 0.5 μL of each primer (20 μM), 0.5 μL of FAM-labeled probe (5 μM), and 3.5 μL of DNase/RNase-free distilled water. Reactions were run on a CFX96 Real-Time PCR thermocycler (Bio-Rad) under the following thermal program: 10 min at 95 °C, followed by 39 cycles of 10 s at 95 °C and 30 s at 60 °C. Samples with a Ct value ≤ 36 were considered positive and were subsequently subjected to conventional PCR targeting the 28S gene (Table 1). Each sample was analyzed in a single qPCR reaction. Positive, negative and extraction controls were included in each run, while DNA quality assessment and PCR inhibition testing were performed as described above (Section 2.4).

**Table 1:** Oligonucleotide sequences of primers and probes used for real-time PCR and conventional PCR in this study.

Target Organism	Target gene	Technique	Name	Primer sequences (5′-3′)	Annealing Temperature	Reference
Bacteria	16S rRNA	Broad-range qPCR	Bact_8FM Bact_515R Bact_338K	AGAGTTTGATCMTGGCTCAG TTACCGCGGCKGCTGGCAC 6FAM- CCAKACTCCTACGGGAGGCAGCAG	60 °C	Palmer et al. (2007)
Vertebrates	Mitochondrial 16S	Broad-range conventional PCR	16SB-260 16SA-2290	CCGGTCTGAACTCAGATCACGT CGCCTGTTTACCAAAAACAT	53 °C	Ficetola et al. (2010)
Kinetoplastida	28S LSU	Broad-range qPCR	F. 24a; 5198	AGTATTGAGCCAAAGAAGG	60 °C	Medkour et al. (2020)
			R. 24a; 5412	TTGTCACGACTTCAGGTTCTAT		
			P. 24a; 5345	FAM- TAGGAAGACCGATAGCGAACAAGTAG		
Kinetoplastida	28S LSU	Broad-range conventional PCR	F2 28S	ACCAAGGAGTCAAACAGACG	58 °C	Medkour et al. (2020)
			R1 28S	GACGCCACATATCCCTAAG		
Ebolavirus	Nucleoprotein	Broad-range qPCR	EBOV_N_MBF	AGACGAGGACGACGAGGAC	60 °C	This study
			EBOV_N_MBR	TTTGAGTTGGCCTGGATTGT		
			EBOV_N_MBP	6FAM- CAAGGGTGGACAACAGAAAAACAGTC		

The PCR used a thermal program of 95 °C for 15 min, followed by 35 cycles of 95 °C for 30 s, annealing temperature for 30 s, and 72 °C for 1 min, with a final extension at 72 °C for 7 min. Amplicons were visualized by 1.5% agarose gel electrophoresis, purified with NucleoFast 96 PCR plates (Macherey-Nagel), and sequenced using the same procedure as above.

### 2.6 Sequencing and phylogenetic analysis

Amplicons from conventional PCR were sequenced using the Big Dye Terminator Cycle Sequencing Kit (PerkinElmer Applied Biosystems) on an ABI automated sequencer (Applied Biosystems). Sequences were assembled and edited using ChromasPro (Technelysium) and compared with GenBank references using BLAST (http://blast.ncbi.nlm.nih.gov/Blast.cgi).

Phylogenetic analyses were performed in MEGA11 using the Maximum Likelihood method with the General Time Reversible (GTR + G) model (Tamura et al., 2021). Initial trees were generated using Neighbor-Joining and BioNJ algorithms. Branch support was assessed by bootstrap analysis (Felsenstein, 1985). Positions with less than 95% site coverage were excluded using the partial deletion option.

**Fig. 2:**
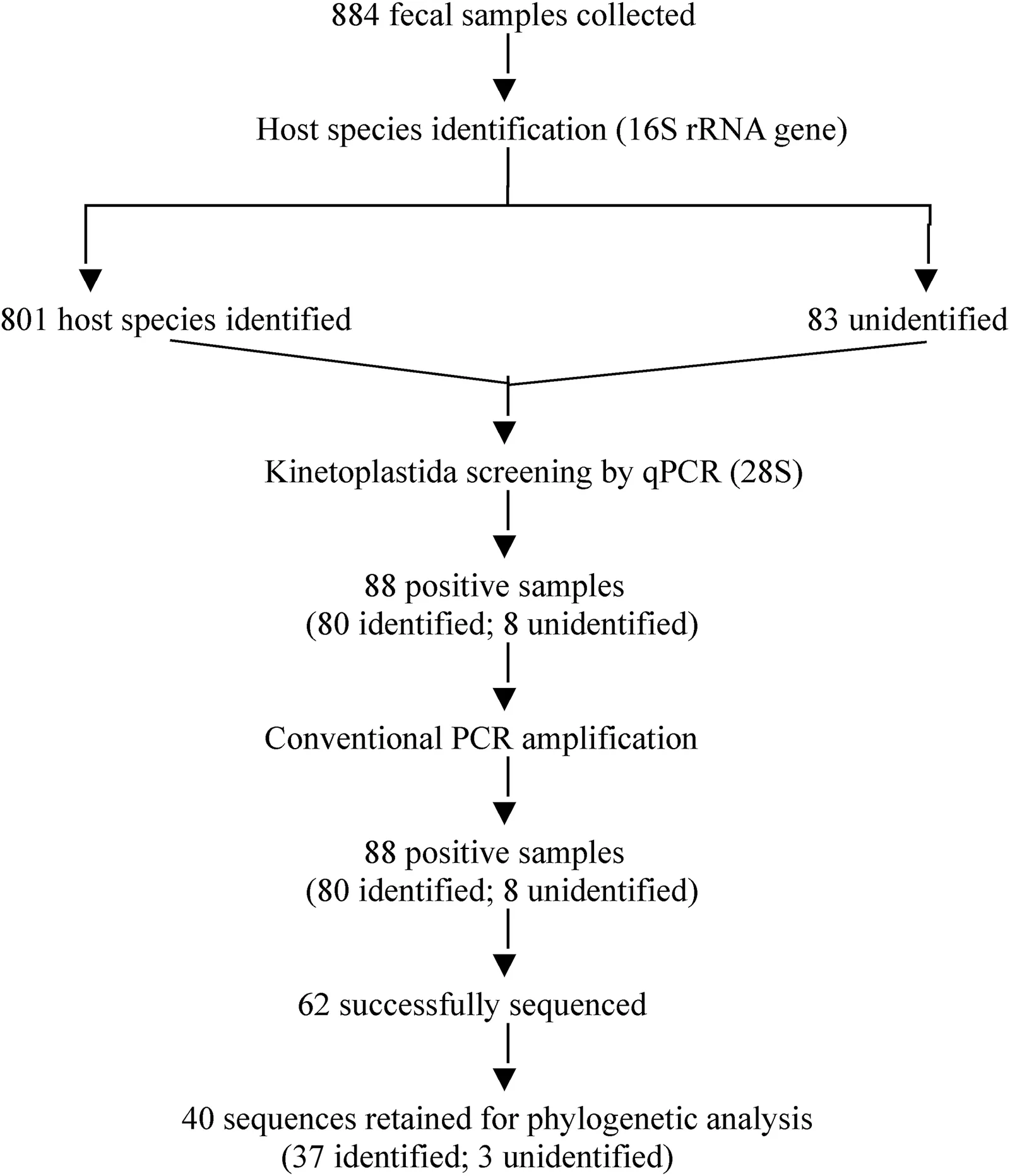
**Workflow of sample processing and molecular analyses.** A total of 884 fecal samples were collected and processed for host species identification and molecular detection of Kinetoplastida. Host species identification based on the 16S rRNA gene was successful for 801 samples, while 83 samples remained unidentified. Screening for Kinetoplastida by qPCR targeting the 28S rRNA gene detected 88 positive samples, including 80 from identified hosts and eight from unidentified hosts. All qPCR-positive samples were successfully amplified by conventional PCR, yielding 62 sequences of sufficient quality for sequence analysis. Of these, 40 sequences were retained for phylogenetic analysis, comprising 37 from identified hosts and three from unidentified hosts*.*

The overall workflow of sample processing and molecular analyses performed in this study is summarized in Fig. 2.

## 3 Results

### 3.1 Sample collection, Ebola virus screening, and DNA quality assessment

The results related to sample collection, Ebola virus screening, and DNA quality assessment have been previously described (Kumakamba et al., 2026). All samples were confirmed negative for Ebola virus and suitable for downstream analyses.

### 3.2 Identification of primate species

Host species identification based on mitochondrial 16S rRNA gene sequencing was successful for 801 of the 884 fecal samples collected (90.6%). The remaining 83 samples could not be assigned to a primate species because of unsuccessful amplification or insufficient sequence quality.

Molecular identification of NHP species from the DRC has been previously described (Kumakamba et al., 2026). Briefly, host species identification was successful for 569 of the 630 samples collected (90.3%). In the RoC, all 117 samples were successfully assigned to species, including *Pan troglodytes troglodytes* (91/117; 77.78%) and *Gorilla gorilla* (26/117; 22.22%), with sequence similarity values ranging from 99.82% to 100%. In Senegal, host species identification was successful for 115 of the 137 collected samples (83.9%), revealing the presence of *P*. *t*. *verus* (78/115; 67.83%) and *Papio papio* (37/115; 32.17%), with sequence similarity values ranging from 99.50% to 100%.

Detailed results, including identified primate species, sequence similarity values and GenBank accession numbers, are presented in Table 2.

**Table 2:** Primate species identified by fecal barcoding.

Country	Primate species identified	Number of samples	Percentage (%)	Similarity with GenBank (%)	Accession Numbers
**DRC**	*Cercopithecus ascanius*	354/569	62.21	97.60-100	AF435520.1; JQ256937.1; JQ256938.1; JQ256940.1; JQ256941.1
	*Cercopithecus nictitans*	1/569	0.18	96.18	JQ256975.1
	*Cercopithecus wolfi*	61/569	10.72	96.19-99.95	JQ256985.1
	*Lophocebus aterrimus*	148/569	26.01	97.19-98.58	KJ434960.1; NC_021954.1
	*Arctocebus aureus*	1/569	0.18	95.23	KC757407.1
	*Allenopithecus nigroviridis*	4/569	0.70	95.79-99.82	NC_023965.1


**Republic of the Congo**	*Gorilla gorilla*	26/117	22.22	100	NC_011120; KF914214.1
	*Pan troglodytes troglodytes*	91/117	77.78	99.82-100	JF727168; KP317203.1; JN191213

**Sénégal**	*Papio papio*	37/115	32.17	99.50-100	MT279064.1; NC_020009.2
	*Pan troglodytes verus*	78/115	67.83	99.66-100	KX211954.1; JF727213.1; JF727208.2

### 3.3 Detection of Kinetoplastida and genetic diversity

Molecular screening targeting the 28S rRNA gene revealed the presence of Kinetoplastida DNA in fecal samples from NHPs across all study sites, with marked differences in detection rates between countries and host species (Table 3).

**Table 3:** Frequency of Kinetoplastida detected in each NHP species by qPCR.

Country	Primate species	Frequency
	*Cercopithecus ascanius* (N = 354)	15 (4.24%)
	*Cercopithecus nictitans* (N = 1)	0 (0%)
	*Cercopithecus wolfi (*N = 61)	5 (8.2%)
**DRC**	*Lophocebus aterrimus* (N = 148)	1 (0.68%)
	*Arctocebus aureus (*N = 1)	0 (0%)
	*Allenopithecus. nigroviridis* (N = 4)	0 (0%)
	Total (N = 569)	**21 (3.69%)**

**Republic of the Congo**	*Gorilla gorilla* (N = 26)	18 (69.23%)
	*Pan troglodytes troglodytes* (N = 91)	1 (1.10%)
	Total (N = 117)	**19 (16.24%)**

**Sénégal**	*Papio papio* (N = 37)	16 (43.24%)
	*Pan troglodytes verus* (N = 78)	24 (30.77%)
	Total (N = 115)	**40 (34.78%)**

In the DRC, 29 samples tested positive. Among these, host species were successfully identified for 21 samples, corresponding to a detection rate of 3.69% (21/569) within the subset of identified hosts. The remaining 8 samples could not be assigned to a specific host species.

Sequences obtained from these unidentified samples were nevertheless included in the phylogenetic analysis, contributing to the overall assessment of Kinetoplastida diversity.

Among identified hosts in the DRC, positive samples were mainly detected in *Cercopithecus ascanius* (15/354; 4.24%) and *C*. *wolfi* (5/61; 8.2%), while only one positive sample was observed in *Lophocebus aterrimus* (1/148; 0.68%). No Kinetoplastida DNA was detected in *C*. *nictitans*, *Arctocebus aureus*, or *Allenopithecus nigroviridis*.

In the RoC, 19 out of 117 samples were positive (16.24%). A high detection rate was observed in *G*. *gorilla* (18/26; 69.23%), whereas only one positive sample was identified in *P*. *t*. *troglodytes* (1/91; 1.10%).

In Senegal, 40 out of 115 samples were positive, corresponding to the highest overall detection rate (34.78%). Positive samples were detected in both species, with higher frequencies in *P*. *papio* (16/37; 43.24%) and *P*. *t*. *verus* (24/78; 30.77%).

**Fig. 3:**
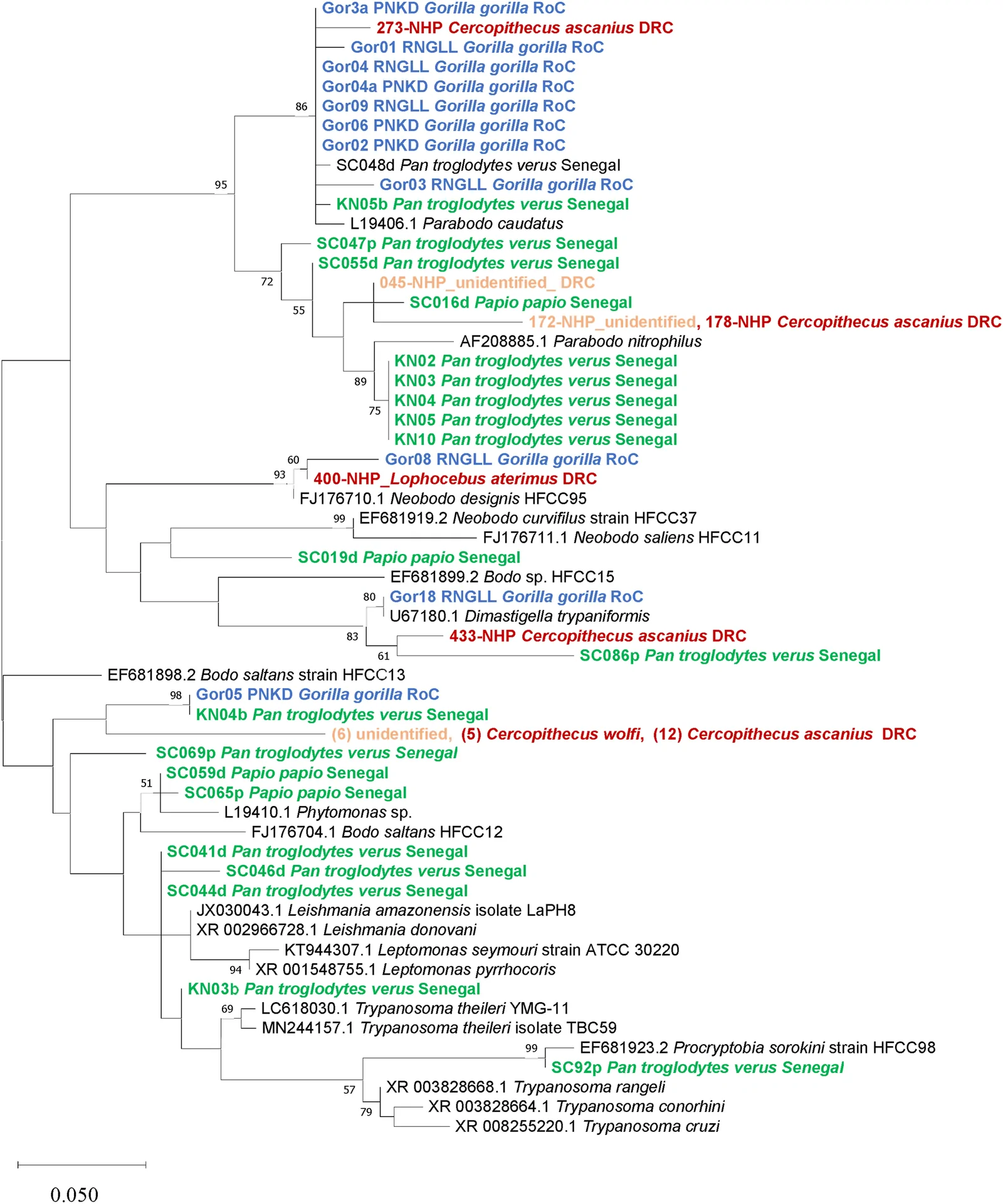
**Phylogenetic tree based on partial 28S rRNA gene sequences of Kinetoplastida detected in non-human primate fecal samples.** The analysis included 58 nucleotide sequences and 839 positions after partial deletion of sites with less than 95% coverage. Bootstrap values obtained from 100 replicates are indicated at branch nodes. The scale bar represents 5% genetic divergence. The sequences shown in blue originate from the Republic of the Congo, those in red and orange from the Democratic Republic of the Congo, while the sequences shown in green originate from Senegal.

All qPCR-positive samples were confirmed by conventional PCR targeting the 28S gene and subsequently sequenced. Phylogenetic analysis based on partial 28S sequences (Fig. 3) revealed that most sequences clustered within bodonid-related clades showing phylogenetic proximity to *Bodo saltans*-like lineages.

This dominant clade included sequences from all three countries, as well as sequences from samples with unidentified hosts, suggesting widespread environmental exposure to bodonid-like kinetoplastids.

In addition, several sequences grouped near *Parabodo nitrophilus* and *Neobodo designis*, indicating the presence of multiple Kinetoplastida lineages. One sequence clustered within a clade closely related to *D*. *trypaniformis*, while another group of sequences formed a clade related to *Parabodo caudatus*. A limited number of sequences also showed phylogenetic proximity to Trypanosomatidae lineages related to Trypanosoma, Leishmania, and Leptomonas. However, several sequences could not be precisely identified at the species level based on the reference sequences currently available. No sequences were identical to parasitic trypanosomatids of medical importance.s.

## 4 Discussion

This study provides evidence of the presence of Kinetoplastida DNA, predominantly belonging to non-trypanosomatid lineages, in fecal samples from wild NHPs across three African countries. Our findings reveal a previously underexplored diversity of kinetoplastids as well as marked geographical variation in detection rates. By combining non-invasive sampling with molecular and phylogenetic approaches, this work contributes to a broader understanding of protist diversity at the wildlife-environment interface.

The frequency of Kinetoplastida DNA detection varied substantially between countries, ranging from 3.69% in the DRC to 16.24% in the RoC and 34.78% in Senegal. Comparisons of detection frequencies among countries should nevertheless be interpreted with caution, as host species, sampling sites, collection periods and sample preservation conditions differed between study areas, precluding assessment of the respective contribution of these factors. Consequently, the observed differences cannot be attributed solely to geographical or biological variation. Comparisons with previous studies remain limited, as data on non-trypanosomatid Kinetoplastida in NHPs are extremely scarce and most available studies rely on blood or tissue samples rather than fecal material (Cordon-Obras et al., 2015; Herder et al., 2002; Medkour et al., 2021b).

At the host level, relatively high frequencies of DNA detection were observed in *G*. *gorilla* in the RoC (18/26; 69.23%), as well as in *P*. *papio* (16/37; 43.24%) and *P*. *t*. *verus* (24/78; 30.77%) in Senegal. However, these proportions should be interpreted with caution, particularly for species represented by relatively small sample sizes. Nevertheless, these findings emphasize the value of investigating understudied NHP species, which warrant further investigation for a better understanding of Kinetoplastida diversity in natural ecosystems.

While studies on kinetoplastids in NHPs have so far mainly focused on parasitic trypanosomatids, our results highlight the presence of bodonid-like lineages in wild primates from several African regions.

Phylogenetic analysis revealed a structured distribution of sequences across multiple kinetoplastid lineages. A large proportion of sequences clustered within a clade closely related to *B*. *saltans*, whereas others were affiliated with *Parabodo nitrophilus* and *Neobodo* species. A smaller subset of sequences showed phylogenetic proximity to Trypanosomatidae lineages, including groups related to Leishmania, Leptomonas, and Trypanosoma. The distribution of sequences across several phylogenetic clades highlights substantial genetic diversity among the Kinetoplastida detected in NHPs. This diversity is consistent with recent literature showing that environmental kinetoplastids represent a phylogenetically and ecologically far more diverse group than initially assumed (D'avila-Levy et al., 2015; Simpson et al., 2006). Molecular approaches, including more recent high-throughput environmental sequencing methods, have notably revealed the existence of numerous bodonid and neobodonid clades that remain insufficiently characterized in aquatic and terrestrial environments (D'avila-Levy et al., 2015; Simpson et al., 2006). These observations suggest that NHPs may be exposed to a much broader diversity of Kinetoplastida than traditionally considered in studies focusing on pathogenic trypanosomatids.

Among the obtained sequences, one sequence detected in *G*. *gorilla* from the RoC (Gor18 RNGLL) was closely related to *D*. *trypaniformis*, while another group of sequences from gorillas, chimpanzees, and one *C*. *ascanius* sample formed a clade related to *B*. *caudatus*. These phylogenetic affiliations are intriguing in light of recent studies reporting the detection of environmental kinetoplastids in clinical samples from vertebrates, including *Parabodo caudatus* in the urine of a dog (Vandersea et al., 2015) and *D*. *trypaniformis* in the urine of a human patient (Peña et al., 2025). However, because samples in our study were collected non-invasively from the environment, no clinical information was available regarding the health status of the primates. Consequently, our data do not allow us to attribute any pathological role to these kinetoplastids in NHPs or to determine the exact nature of their association with primate hosts.

The repeated detection of these kinetoplastids in several NHP species and across different environments nevertheless suggests that wild NHPs are repeatedly exposed to these organisms in their natural habitats. However, the currently available data do not allow us to determine whether these detections reflect simple transient passage following environmental exposure or a more persistent association with primate hosts. Further investigations will be necessary to better understand the nature of these interactions.

No clear evidence of medically important trypanosomatids was identified, although some sequences showed phylogenetic proximity to Trypanosomatidae lineages. This is consistent with the biology of pathogenic Trypanosomatidae, which are mainly blood- or tissue-dwelling parasites transmitted by hematophagous vectors (Stuart et al., 2008; Szöke et al., 2017).

One sequence (KN03b) showed phylogenetic proximity to *T*. *theileri*, whereas three other sequences (SC041d, SC044d, and SC046d) were related to lineages close to Leishmania and Leptomonas. Such observations remain rare in fecal samples from NHPs. For comparison, a study conducted in neotropical primates reported the detection of *Trypanosoma* spp. in only 2 out of 76 analyzed fecal samples (Carrillo-Bilbao et al., 2022). Our findings therefore deserve further investigations to clarify their biological significance, given that Trypanosomatidae are usually detected in blood, tissues, or their vectors.

Although repeated collection from the same location was avoided and successive fecal samples were collected at spatially separated points to minimize the likelihood of sampling the same individual, individual identification of the primate from which each fecal sample originated was not possible in the field; therefore, repeated sampling of the same individual cannot be completely excluded.

## 5 Conclusion

This study demonstrates the presence of diverse Kinetoplastida DNA in fecal samples from wild NHPs across three African regions, with a predominance of non-trypanosomatid lineages. Phylogenetic analyses revealed multiple kinetoplastid affiliations, including bodonid-like lineages related to Bodo, Parabodo, and Dimastigella, highlighting substantial genetic diversity among the detected Kinetoplastida.

The detection of a limited number of sequences related to Trypanosomatidae, including lineages close to Trypanosoma, Leishmania, and Leptomonas, represents an uncommon observation in fecal samples from NHPs. However, DNA detection alone does not demonstrate active infection or host colonization, and the biological significance of these findings remains unclear, particularly because Trypanosomatidae are usually detected in blood, tissues, or their vectors.

Overall, these results highlight the value of non-invasive sampling approaches for exploring overlooked components of eukaryotic microbial diversity and provide new insights into the diversity and occurrence of environmental Kinetoplastida in African ecosystems. Further studies combining complementary sample types, strain isolation, and additional molecular approaches will be necessary to better understand the ecological significance of the detected lineages and their relationships with vertebrate hosts.

## CRediT authorship contribution statement

**Charles Kumakamba:** Writing – review & editing, Visualization, Resources, Methodology, Investigation, Data curation. **Inestin Amona:** Writing – review & editing, Resources, Methodology, Investigation, Data curation. **Clément Labarrere:** Writing – review & editing, Visualization, Methodology, Investigation, Data curation. **Aku Elomou Essenou:** Writing – review & editing, Investigation. **Georges Diatta:** Writing – review & editing, Investigation. **Cheikh Sokhna:** Writing – review & editing. **Jean Akiana:** Writing – review & editing. **Jean-Jacques Muyembe-Tamfum:** Writing – review & editing. **Florence Fenollar:** Writing – review & editing, Validation, Supervision, Resources, Project administration. **Oleg Mediannikov:** Writing – review & editing, Validation, Supervision, Resources, Project administration, Methodology, Investigation, Conceptualization.

## Ethical approval and consent to participate

In the DRC, the collection of NHP samples was conducted in accordance with ethical regulations and authorized by the Center for Research in Ecology and Forestry (CREF), Mabali, under the approval letter No. 0051/MIN.R.S.I.T./CREF/MAB/DG/01/MMIK/2023, issued on 17 November 2023.

In Senegal, research authorization was issued by the Ministry of Environment and Ecological Transition (No. 003924/DEFCCS/DGF, December 4, 2020).

In the RoC, research and administrative authorizations were granted by the Ministry of Scientific Research and Technological Innovation (Nos. 003/MRSIT/DGRST/DMAST and 05/MRSIT/DGRST/DMAST) and by the Congolese Agency for Wildlife and Protected Areas (ACFAP) (No. 00087/ACFAP/DG/DTS-SRM).

## Consent for publication

Not applicable.

## Availability of data and materials

The sequence assemblies generated in this study have been deposited in GenBank and are available under the following accession numbers: PZ490969, PZ490970, PZ490971, PZ490972, PZ490973, PZ490974, PZ490975, PZ490976, PZ490977, PZ490978, PZ490979, PZ490980, PZ490981, PZ490982, PZ490983, PZ490984, PZ490985, PZ490986, PZ490987, PZ490988, PZ490989, PZ490990, PZ490991, PZ490992, PZ490993, PZ490994, PZ490995, PZ490996, PZ490997, PZ490998, PZ490999, PZ491000, PZ491001, PZ491002, PZ491003, PZ491004, PZ491005, PZ491006, PZ491007, PZ491008, PZ491009, PZ491010, PZ491011, PZ491012, PZ491013, PZ491014, PZ491015, PZ491016, PZ491017, PZ491018, PZ491019, PZ491020, PZ491021, PZ491022, PZ491023, PZ491024, PZ491025, PZ491026, PZ491027, PZ491028, PZ491029, PZ491030.

## Funding information

This work was supported by the Institut Hospitalo-Universitaire (IHU) Méditerranée Infection and the French Government managed by the National Research Agency under the “Investissements d'avenir (Investments for the Future)” programme with the reference ANR-10-IAHU-03 (Méditerranée Infection), by the Contrat Plan Etat-Région and the European funding FEDER IHUPERF.

## Conflict of interests

The authors declare that there are no conflicts of interest.
